# Pharmacological Properties of Shionone: Potential Anti-Inflammatory Phytochemical against Different Diseases

**DOI:** 10.3390/molecules29010189

**Published:** 2023-12-28

**Authors:** Varun Jaiswal, Hae-Jeung Lee

**Affiliations:** 1 Department of Food and Nutrition, College of BioNano Technology, Gachon University, Seongnam 13120, Republic of Korea; computationalvarun@gmail.com; 2 Institute for Aging and Clinical Nutrition Research, Gachon University, Seongnam 13120, Republic of Korea; 3 Department of Health Sciences and Technology, GAIHST, Gachon University, Incheon 21999, Republic of Korea

**Keywords:** NF-κB, shionone, inflammation, cancer, therapeutic, supplement, triterpenoid, Parkinson’s disease

## Abstract

Shionone is a triterpenoid that is the primary constituent of an important ancient Chinese medicine named Radix Asteris. It has emerged as an attractive candidate against different important diseases, including interstitial cystitis, colitis, cancer, Parkinson’s disease, and urinary tract infections, and was found to have a protective effect on multiple organs, including the colon, kidneys, lungs, brain, and bladder. The anti-inflammation activity of shionone may be considered an important property that imparts the positive health outcomes of shionone. Important molecular targets and markers such as TNF-α, STAT3, NLRP3, and NF-κB were also found to be targeted by shionone and were verified in different diseases. This suggests the possible potential of shionone against other diseases associated with these targets. Pharmacokinetic studies also support the therapeutic potential of shionone and provide the initial track that may be pursued for its development. Yet, the compilation of the pharmacological activities of shionone and its important genes and pathway targets are absent in the existing literature, which would direct its development as a therapeutic and/or supplement. Hence, the present review provides a compilation of information concerning pharmacological activities, highlights the existing holes, and proposes a specific direction for the expansion of shionone as a therapeutic against different diseases and conditions.

## 1. Introduction

Shionone is one of the major constituents of Radix Asteris (RAS), which is the dried rhizome and root of the plant *Aster tataricus* (AT) [1]. RAS has been used for the treatment of cough and phlegm since early times in ancient Chinese medicine (ACM). Recent studies also showed several other important pharmacological properties such as anti-inflammatory, antioxidant, antitumor, and anti-depression effects [1]. Shionone is one of the important triterpenoid components of phytochemicals present in AT, which is considered a content determination index for RAS quality and is believed to be primarily responsible for its pharmacological properties. Triterpenoids are important phytochemicals known to possess a variety of important medicinal properties that can make them useful as therapeutic agents [2,3].

Considering its importance, shionone has been studied for different pharmacological properties including anti-inflammatory, antioxidant, anti-cancer, anti-colitis, anti-chronic obstructive pulmonary disease (COPD), and protective effects on the lungs, kidneys, colon, brain, and bladder. Inspired by these pharmacological properties of shionone, researchers further unveiled the possible action mechanisms through different targets and pathways. In these studies, shionone was found to target important key targets, such as TNF-α, STAT3, NLRP3, STAT5, and NF-κB, which suggests that the role of shionone may be analyzed against other diseases linked with these crucial drug targets [4,5,6,7,8]. Considering the pharmacological importance and presence of a rare 6/6/6/6 tetracyclic skeleton with a C-3 carbonyl carbon in shionone, researchers have been working on its synthesis [9,10,11]. The biosynthesis of shionone was also studied, and researchers have discovered the oxido squalene cyclase from AT, which can produce a high yield of shionone; they designated it as shionone synthase [11]. In various pharmacological studies, the anti-inflammatory effect of shionone, especially the inhibition of TNF-α, NLRP3, and NF-κB, was observed, which suggested that it may be considered an important property behind the protective role of shionone on different organs and diseases. However, the compiled information regarding the pharmacological properties, mechanism of action, and important targets of shionone is absent in the existing literature, which could serve as a resource to help the expansion of shionone as a therapeutic/supplement against important diseases. Hence, the current study not only compiles the pharmacological properties of shionone but also discusses the possible mechanism along with the proposed direction of future research for the development of shionone as a supplement and/or therapeutic candidate against important diseases and conditions, including cancer.

## 2. Literature Search for Shionone

A literature search was conducted using keywords related to the study, including shionone and its combinations with disease, pharmacological activity, anti-cancer, pharmacology, anti-inflammatory, in vivo, and in vitro studies. Important online databases considered in the literature search were PubMed, Google Scholar, Scopus, Google, and ResearchGate. Patents were also searched with the same keywords through a Google and Espacenet patent search. In keyword searches, 48 unique articles and 10 patents were retrieved from the search hits and considered for further study. Search hits from the keywords include research and review articles, patents, and book chapters available in the English language until September 2023, which were incorporated, and the relevant literature as per the study topic was included in the manuscript.

## 3. Structure and Properties of Shionone

**Figure 1:**
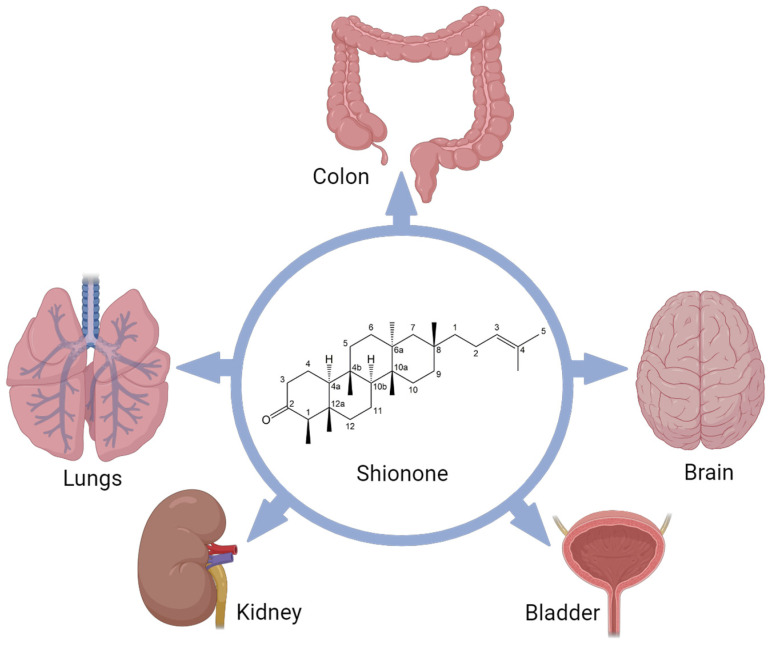
Protective role of shionone in multiple organs in in vivo studies. “Created with BioRender.com accessed on 18 December 2023”.

Shionone is a tetracyclic triterpenoid that has an oxo group, a 4-methyl-3-pentenyl sidechain, and six methyl groups. It can be referred to as (1*R*,4a*S*,4b*S*,6a*S*,8*R*,10a*R*,10b*S*,12a*S*)-1,4b,6a,8,10a,12a-hexamethyl-8-(4-methylpent-3-en-1-yl) hexadecahydrochrysen-2(1*H*)-one (Figure 1). It is isolated from the roots and rhizomes of *Aster tataricus* (AT) and it has also been reported from the roots of other plant species, such as *Ligularia fischeri* and *Millettia speciosa* [12,13,14]. The content of shionone in plants may vary, as per the local environment. The content of shionone in the dried roots and rhizome of AT (Radix Asteris) is reported to be between 0.026 and 0.41%. The molecular weight of shionone is 426.7 g/mol, and it has a hydrogen bond acceptor. A limited toxicity study has been conducted on shionone. In cell line experiments, no toxicity in shionone was observed at 20 µg/mL on SV-HUC-1 cells [15], and in different animal studies, up to a 200 mg/kg treatment dose has been used on rats in disease models [15,16].

## 4. Pharmacological Activities of Shionone

Initial studies of shionone were conducted according to the pharmacological properties of parent AT extract followed by the analysis of possible targets. These studies may be divided according to activities and type of experiments, like in silico, in vitro, and in vivo experiments.

### 4.1. Lung Protective and Anti-COPD Activities of Shionone

Three lung diseases (chronic obstructive pulmonary disease (COPD), lower respiratory tract infections, and lung cancer) are among the top six diseases responsible for death worldwide [17]. Natural products have been suggested for lung protective activities in traditional medicine [18]. The lung protective activities of shionone were studied, as RAS has been used to treat cough and phlegm in ACM. The different in vitro and in vivo models challenged through different toxins and conditions were studied for lung protection by shionone [19,20,21].

#### 4.1.1. In Vitro Lung Protective Activities of Shionone

In different studies, the lung protective effect of shionone was analyzed by a combination of in vitro and in vivo experiments. In a recent study, the lung cell line A549 was utilized to study the protective effect of shionone against pneumolysin, which is the toxin produced by *Streptococcus pneumoniae*. The result revealed that shionone at 8 μg/mL could alleviate A549 cell injury by pneumolysin activity. It was also found that shionone significantly inhibited the hemolytic activity of pneumolysin at 4 μg/mL [19]. In further experiments, it was observed that shionone inhibits the toxicity of pneumolysin by inhibiting the oligomerization of pneumolysin in a dose–dependent manner. However, shionone did not inhibit the expression of pneumolysin at 4 μg/mL [19]. The minimum inhibitory concentration of shionone against *Streptococcus pneumoniae* was found to be 128 μg/mL in the patent study [22]. In another study, the lung protective effects and mechanism of shionone were explored on the murine RAW264.7 macrophage cells with animal studies. The protective effect of shionone against lipopolysaccharide (LPS)-induced inflammation was observed in different experiments. The treatment of shionone reduced the supernatant levels of TNF-α, IL-1β, and IL-6 [20]. Similarly, an obvious increase in the expressions of CD16/CD32 and CD206 through LPS exposure compared to the control group was observed. Shionone treatment reduced the expression of CD16/CD32 and iNOS and increased the expression of CD206. Additionally, shionone treatment also increased the expression of Arg1 [20]. These studies suggested that the inhibition of pneumolysin and the reduction in inflammation may be the main reason behind the protective effect of shionone on lungs and lung cell lines against pneumolysin and LPS. In a recent study, considering the anti-COPD activity of Bufei Yishen granules (BYGs) in traditional Chinese medicine, a multi-omic study has been designed to discover important components and their targets. In the study, integrated multi-omics analysis, including molecular networking, metabonomics, and bioinformatics, revealed the key components and targets, which include the epidermal growth factor receptor (EGFR) targeted by shionone. As myosin light chain 2 (MLC2) is a diagnostic marker in COPD and the downstream protein of the EGFR pathway, the cigarette smoke extractive (CSE)-induced BEAS-2B cell model of MLC2 phosphorylation was studied. Shionone significantly suppressed the phosphorylation of MLC2 in a dose–dependent manner in the Western blot experiment (Table 1). The findings of the study suggested that shionone may exert the anti-COPD effect by delaying the airway remodeling process by targeting EGFR [21].

#### 4.1.2. In Vivo Lung Protective Activities of Shionone

**Figure 2:**
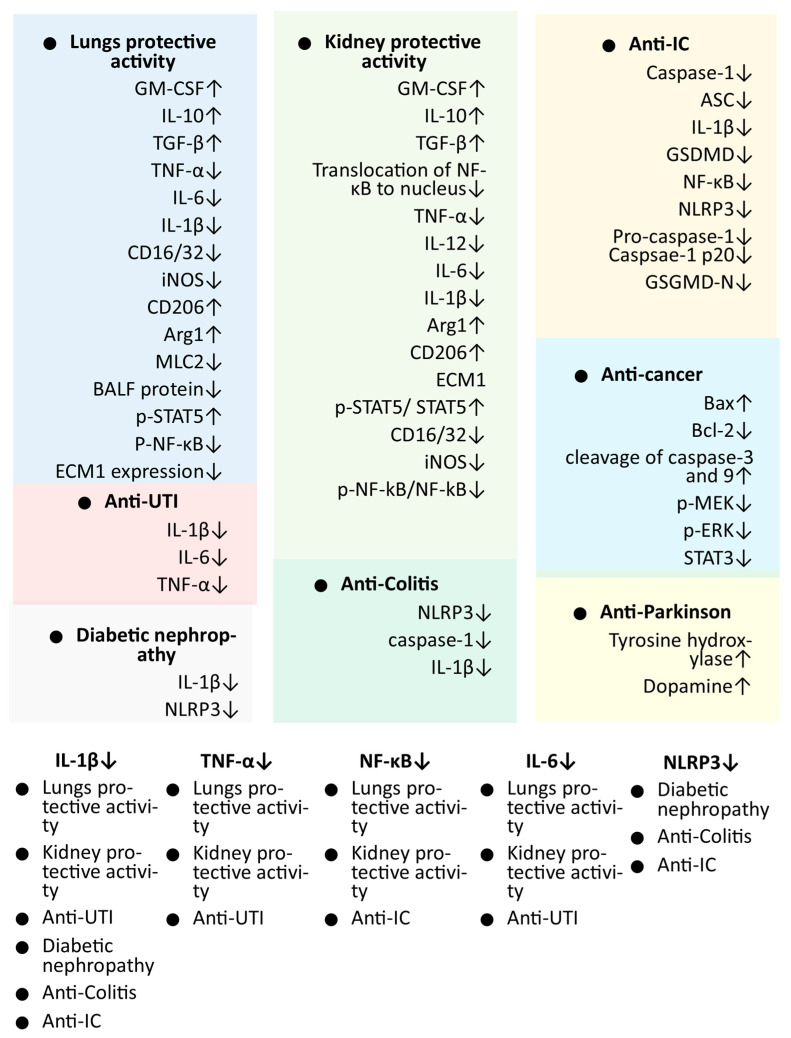
Markers/targets associated with the pharmacological activities of shionone. Genes identified in in vivo studies and found to be associated in more than two studies are listed with a white background. Abbreviation: Anti-IC: interstitial cystitis; Anti-UTI: urinary tract infection; Arg1 arginase 1; ASC apoptosis-associated speck-like protein containing a CARD; BALF: bronchoalveolar lavage fluid; Bax: BCL2-associated X; Bcl-2 B-cell lymphoma 2; CD: cluster of differentiation; ECM1: extracellular matrix protein 1; GM-CSF granulocyte–macrophage colony-stimulating factor; GSDMD: gasdermin D; IL: interleukin; iNOS: inducible nitric oxide synthase; MLC2: myosin regulatory light chain 2; NF-κB: nuclear factor-κB; NLRP3: NOD-, LRR-, and pyrin domain-containing protein; p-ERK: phosphorylated extracellular signal-regulated kinase; p-STAT5: phosphorylated signal transducer and activator of transcription; TGF-β: transforming growth factor β; TNF-α: tumor necrosis factor α; ↑: up-regulation; ↓: down-regulation.

The lung protective effect of shionone was studied in different mouse models. Female BALB/c mice were utilized as mouse pneumonia models after intranasal infection (4 × 10^8^ CFU/mouse) with *Streptococcus pneumoniae*. Damage to the lung tissue was observed in the animals after the infection compared to the control group, which was improved with shionone treatment. Similarly, a histopathology study of lung tissue revealed distorted alveolar structure and infiltration of inflammatory cells in the infection group compared to the control. Conversely, in the shionone treatment group, a reduction in infiltration of inflammatory cells and intact alveolar structure was observed. Additionally, colonies of *Streptococcus pneumoniae* were also reduced in the shionone treatment group by 4 × log10 compared with the model (infections) group [19]. In another study on male ICR mice, cecal ligation and puncture surgery (CLPS) was used to induce the sepsis model. Within 12 h, all mice in the CLPS group died, but shionone treatment significantly improved the survival of mice at a 100 mg/kg concentration [20]. Compared to the sham (control) group, the CLPS group has shown histopathological alteration through Hematoxylin and eosin (H&E) staining, which includes the thickening of alveolar wall, the infiltration of inflammatory cells, and pulmonary edema. These alterations were alleviated in shionone treatment and Dexamethasone (positive control) treatment groups in the study [20]. Further, the expression of BALF protein and lung MPO activity was significantly reduced in the shionone treatment and was found to be elevated in the CLPS group. Similarly, serum levels of TNF-α, IL-6, and IL-1β were significantly suppressed in the shionone treatment and were found to be elevated in the CLPS group. In the flow cytometry analysis, it was also observed that the CLPS remarkably enhanced the neutrophils and macrophages ratio in the whole population of BALF cells while in the shionone treatment group, a reduced cell population percent of the neutrophils and macrophages was observed [20]. Further, the macrophage study revealed that the M1 polarization indicators CD16/32 and iNOS were increased in the CLPS group and were reversed in the shionone treatment group [20], while the M2 polarization biomarkers, CD206 and Arg1, were upregulated in the shionone treatment group. In the ECM1/STAT5/NF-κB pathway study, the enhancement in phosphorylation of STAT5 and the inhibition of ECM1 expression and the phosphorylation of NF-κB was observed in the shionone treatment group (Table 2 and Figure 2). Shionone treatment may inhibit the M1 macrophage polarization through the ECM1/STAT5/NF-κB pathway and enhance M2 macrophage polarization [20].

#### 4.1.3. In Silico Docking of Shionone with Pneumolysin

A study has suggested that the inhibition of oligomerization of pneumolysin is the key step for the lung protective activity of shionone [19]. Hence, to further study the site of interaction and binding of shionone with pneumolysin, a molecular docking simulation was conducted. Molecular docking is the robust and widely used computational method to study protein–ligand interactions [23,24]. The important residues of pneumolysin such as ASP-59, ILE-60, THR-57, PHE-344, and ASN-346 were involved in the interaction with shionone in the docked complex. The docking result of binding affinities and inhibitor constant of shionone were found to be 12.9 kcal/mol and 0.342 nM, respectively, in shionone and pneumolysin binding [19].

### 4.2. Kidney Injury Protection through Shionone

Acute kidney injury is a serious complication that is associated with a high mortality rate [25]. Considering the anti-inflammatory effect of shionone and the kidney protective potential of natural compounds, the kidney protection activity of shionone was also discovered in cell lines and animal model experiments [26,27].

#### 4.2.1. In Vitro Kidney Protective Activity of Shionone

RAW264.7 macrophage cells were used to study the mechanism of kidney protection through inhibition of inflammation-related parameters. LPS enhanced the nucleus translocation of NF-κB, as the intensity ratio of the nucleus/cytoplasmic increased with LPS incubation. The translocation of NF-κB to the nucleus from the cytoplasm was reduced in a dose–dependent manner with shionone treatment, and the intensity ratio of the nucleus/cytoplasmic was found to be reduced. The expression of inflammation-related factors such as IL-6, IL-1b, IL-12, TNF-α, CD16/32, iNOS, and p-NF-kB/NF-kB was downregulated in the shionone treatment cells and was found to be upregulated due to the LPS incubation. Similarly, the expression of ECM1 decreased with the treatment of shionone in dose–dependent manner. The expression of Arg1, CD206, and p-STAT5/STAT5 were found to be enhanced due to shionone treatment [26].

#### 4.2.2. In Vivo Kidney Protective Activity of Shionone

A mouse model of sepsis-induced acute kidney injury was studied for the kidney protective activity of shionone. Cecum ligation and puncture surgery (CLPS) were conducted on C57BL/6 mice to create the sepsis-induced acute kidney injury model [26]. Different concentrations (50 and 100 mg/kg) of shionone were orally administered to mice, and the Dexamethasone drug was used as positive control in the experiments. Shionone treatment reduced the inflammatory cell infiltration and vacuolation in the histopathology analysis, which increased due to kidney injury in the CLPS group. Shionone treatment also improved the serum anti-inflammatory parameters, like a reduction in inflammatory factors IL-6, IL-1b, IL-12, and TNF-α, which were enhanced in the CLPS group [26]. Further, shionone treatment neutralized the enhanced expression of CD16/32, iNOS, and p-NF-kB/NF-kB in the CLPS group. A reduction in the expression of ECM1 was also found to be significant in both the shionone treatment and positive control groups. Similarly, shionone and positive control treatments were found to enhance the expressions of CD206, Arg1, and p-STAT5/STAT5 [26]. Shionone treatment was also found to improve renal function parameters, such as blood urea nitrogen and serum creatinine [26]. In another study, the anti-diabetic nephropathy activity of shionone was studied in a mouse model. C57BL/6 wild type mice were used to induce diabetic nephropathy through a single nephrectomy and streptozotocin administration [28]. Shionone treatment resulted in improvement in the body weight, kidney index, and local inflammation of glomeruli. Reduction in inflammatory factors, such as IL-1b and NLRP3, was also observed in the immunohistochemistry study [28]. The study revealed the protective and therapeutic role of shionone against diabetic nephropathy with the reduction in inflammation, which highlights its potential to develop as a therapeutic candidate [28]. Further studies with other models may be suggested to establish the anti-diabetic nephropathy activity of shionone.

### 4.3. Anti-Cancer Activity of Shionone

Anti-inflammation and possible antioxidant activities of shionone inspired researchers to explore its anti-cancer activity, as these activities can contribute to the anti-cancer properties of a compound [29,30,31]. One of the important cancers i.e., breast cancer [32,33], was selected to study the anti-cancer effects of shionone through normal breast cell lines and breast cancer cell lines, which were MB-157 and SK-BR-3 cell lines, respectively [34]. Shionone was able to inhibit the breast cancer cells (SK-BR-3) selectively and exerted a limited effect on normal breast cells (MB-157). Shionone application caused the deformation of the nucleus in the breast cancer cells, which was higher at higher concentrations. Shionone increases the expression of Bax and causes the cleavage of caspase-3 and -9, which may be a reason for apoptotic activity observed in the breast cancer cells. It also decreases the expression of bcl-2 in a dose–dependent manner. Along with selective inhibition of breast cancer cells, it also inhibited the migration and invasion of breast cancer cells by 25% and 37%, respectively, at a 24 μM concentration [34]. Additionally, the effect of shionone on the STAT3 and MEK/ERK signaling pathways was also studied by Western blot analysis, and the phosphorylation of STAT3, MEK, and ERK was found to be decreased in dose–dependent manners, which clearly indicates that these may be the target pathways for the anti-breast cancer activity of shionone [34].

**Table 1:** In vitro pharmacological activities of shionone.

Activity	Dose	Method	Result	References
Lung protective and anti-COPD activities	4–32 μg/mL	Hemolysis test of pneumolysin	Pneumolysin hemolytic activity↓	[19]
		WB	Expression of pnemolysin↓	
		A549 cell injury of pneumolysin through live/dead assays	A549 cell injury↓	
	2, 4, and 8 μg/mL	WB and optical density analysis	Oligomerization of pneumolysin↓	
	0.5, 1.0, and 2.0 μg/mL	LPS-induced RAW264.7 cells and ELISA	Level of IL-10↑, GM-CSF↑, TGF-β↑, TNF-α↓, IL-6↓, and IL-1β↓	[20]
		LPS-induced RAW264.7 cells and a fluorescence microscope	CD16/32↓, iNOS↓ (M1 polarization indicators), CD206↑, and Arg1↑ (biomarkers of M2 polarization)	
	1 and 10 μM	CSE-stimulated BEAS-2B cell model of MLC2 and WB	MLC2↓	[21]
Kidney protective activity	0.5, 1.0, and 2.0 μg/mL	RAW264.7 cells treated with LPS, cell viability by MTT, and immunofluorescence staining for markers	Cell viability↑, level of GM-CSF↑, IL-10↑, IL-12↓, IL-6↓, TGF-β↑, TNF-α↓, and IL-1β↓	[26]
		WB	Arg1↑, CD206↑, ECM1, and p-STAT5/STAT5↑	
		LPS-induced RAW264.7 cells and immunofluorescence	Translocation of NF-κB to the nucleus↓	
Anti-cancer activity	3.12–100 μM	SK-BR-3 breast cancer cell proliferation studied with a CCK assay	Growth of breast cancer cells↓ (IC_50_ = 14 μM)	[34]
	0, 7, 14, and 28 μM shionone	Nuclear morphology by DAPI staining and fluorescent microscopic examination of the AO/EB-stained cells	Nuclear deformation↑	
		WB analysis	Bcl-2↓, cleavage of caspase-3 and -9↑, and Bax↑	
		Transwell assay for migration and invasion	Migration↓ and invasion↓	
		WB	p-MEK↓, p-ERK↓, and STAT3↓	
Anti-IC	2.5, 5, and 10 μg/mL	MTT, Hoechst33342, and PI double staining	Cell viability↑ and PI-positive cell rates↓	[16]
	SV-HUC-1 cell	RT-PCR	NLRP3↓, caspase-1↓, ASC↓, IL-1β↓, and GSDMD↓	
		WB and ELISA	NF-κB↓, NLRP3↓, pro-caspase-1↓, caspsae-1 p20↓, and GSDMD and GSGMD-N↓	
Anti-UTI activity	5, 10, and 20, μg/mL	Study the bacterial growth in SV-HUC-1 cells through cell smear plate experiments and immunofluorescence	CFU of bacteria↓	[15]
		ELISA	TNF-α↓, IL-1β↓, and IL-6↓	

AO/EB, acridine orange/ethidium bromide; CCK, cell counting kit; CFUs, colony forming units; COPD, chronic obstructive pulmonary disease; ELISA, enzyme-linked immunosorbent assay; CSE, cigarette smoke extractive; IC, interstitial cystitis; LPS, lipopolysaccharide; MTT, 3-(4,5-dimethylthiazol-2-yl)-2,5-diphenyltetrazolium bromide; PI, propidium iodide; RTPCR, reverse-transcriptase polymerase chain reaction; UTI, urinary tract infection; WB, Western blot; ↑: up-regulation; ↓: down-regulation.

### 4.4. Anti-Interstitial Cystitis Activity of Shionone

Interstitial cystitis (IC), also known as bladder pain syndrome, is a chronic syndrome that may cause urgent urination, frequent urination, and pain in the bladder or pelvis. It can seriously distress the physical and mental health of patients, and women are more susceptible than men. Limited symptomatic treatment options are only available for IC. Hence, research on effective treatment is highly required for IC. Recently, plant-based therapeutics have shown promising results against IC [16,35]. The anti-IC activity of shionone was studied in both in vitro and in vivo experiments.

#### 4.4.1. In Vitro Anti-IC Activities of Shionone

To study the anti-IC effect of shionone and the possible mechanism of action, the bladder epithelial cell line (SV-HUC-1) was utilized. Shionone was able to increase the cell viability of SV-HUC-1 cells in a dose–dependent manner, which decreased with the application of LPS and ATP, and the results were consistent in 3-(4,5-dimethyl-2-thiazolyl)-2,5-diphenyl-2-H-tetrazolium bromide (MTT) and staining experiments (Table 1). Further, the study revealed the expression of pyroptosis-related genes such as GSDMD, NLRP3, caspase-1, ASC, and IL-1β was found to be decreased in a dose–dependent manner with shionone treatment, which increased due to LPS and ATP application. Additionally, in shionone treatment, the protein expression level of GSDMD, GSGMD-N, NLRP3, caspase-1, pro-caspase-1, p20, and NF-κB was found to be downregulated compared to the LPS- and ATP-administered groups. Additionally, the concentration of IL-1β and the activity of caspase-1 were also found to be downregulated with shionone treatment in a dose–dependent manner in ELISA and caspase-1 colorimetric assays, respectively [16].

#### 4.4.2. In Vivo Study of the Anti-IC Activity of Shionone

Female Sprague–Dawley (SD) rats were used to study the activity of shionone against interstitial cystitis (IC). Cyclophosphamide was used to create the model of IC and bladder injury. A sodium 2-mercaptoethanesulfonate drug (mesna) was used as a positive control in the study [16].

Bladder wet weight, edema score, hemorrhage score, and histopathological score were significantly reduced with shionone treatment in a dose–dependent manner, which was comparable with the mesna (positive drug group) group. The results conspicuously demonstrated a reduction in inflammation with shionone and drug treatment groups, which occurred due to the cyclophosphamide [16]. Further, the expression of pyroptosis-related proteins in the animals such as NF-κB, GSGMD-N, NLRP3, ASC, pro-caspase-1, and cleaved IL-1β decreased significantly in shionone treatment groups in a dose–dependent manner compared with saline groups. Additionally, the expression of NLRP3 was also studied through the immunofluorescence method, which confirmed a reduction in NLRP3 fluorescence compared with the saline group. The findings suggest the suppression of NLRP3 inflammasome with shionone treatment, which was an activated animal model in the study [16].

### 4.5. Activity of Shionone against Urinary Tract Infections

A urinary tract infection (UTI) is one of the most common infectious diseases, accounting for more than 400 million cases and 236,786 deaths globally in 2019 [36]. The increasing trend of UTI cases worldwide and antibiotic resistance ignites the demand for new interventions for the disease. Herbal sources are one of the suggested sources for the discovery of a new drug candidate that may be effective against drug resistance [37,38]. Shionone was studied for its potential against UTI through an uropathogenic strain of *Escherichia coli* (UPSEC) in a UTI model of SD rats and SV-HUC-1 cells.

#### 4.5.1. Anti-UTI Activity of Shionone in an In Vitro Study

SV-HUC-1, the human bladder epithelium cells, were used to assess the anti-UTI effect of shionone. Initially, the different concentrations of shionone (0.1, 1, 2.5, 5, 10, 20, 40, and 80 μg/mL) were utilized in the MTT assay to select the treatment doses. The in vitro cell line model was utilized through bacterial infection (*E. coli* UTI89) in SV-HUC-1 cells in a 1:10 ratio. Fluorescent staining showed that the shionone treatment effectively reduced the amount of *E. coli*, which was present in large amounts in UPSEC groups. Similar results were observed in the cell smear plate study, and the colony-forming unit of bacteria was negatively correlated with the treatment concentration of shionone [15]. Furthermore, the expression of inflammatory factors such as IL-1β, IL-6, and TNF-α was also reduced with shionone treatment in a dose–dependent manner, which was elevated due to UPSEC [15].

#### 4.5.2. Anti-UTI Activity of Shionone in an In Vivo Model

The anti-UTI effect of shionone was studied against SD rats challenged with an uropathogenic *E. coli* strain. Two doses of shionone (100 mg/kg and 200 mg/kg) and the positive control drug levofloxacin were provided to the animals through oral gavage [15]. Like the previous study, shionone improved the congestion and edema in the bladder of rats in the shionone treatment group compared with the saline group. Similarly, bladder injuries were significantly reduced in the shionone and positive drug treatment groups, which were induced through UPSEC and seen as infiltrations of inflammatory cells in large submucosal and mucosa thickening of the bladder. Additionally, electron microscopy of superficial cystic tissue cells revealed that the lysosomes and vesicles improved in the shionone treatment group and significantly reduced due to UPSEC. Shionone also effectively reduced the presence of *E. coli* in bladder tissue at both treatment concentrations. Further, inflammatory markers such as IL-1β, IL-6, and TNF-α are known to be elevated in the UTI patients and were also significantly reduced with shionone treatment and were found to be elevated in the UPSEC group. The study suggested that the anti-UTI effect of shionone may be due to inhibition of *E. coli* and inflammation [15].

### 4.6. Antioxidant Activity of Shionone

A variety of compounds have been isolated from the plant *Aster tataricus* (AT), and shionone is the major triterpenoid compound found in the plant. Considering the different pharmacological properties of the plant, the antioxidant properties of different compounds from the extract of AT were studied. The antioxidant properties of these compounds were observed in different methods, including the inhibition of lipid peroxidation, the inhibition of erythrocyte hemolysis, and superoxide radical scavenging. The most active antioxidant compounds in these assays from AT were quercetin and kaempferol. Performance of shionone in terms of the inhibition of hemolysis of rat erythrocytes, lipid peroxidation, and superoxide radical generation were 11.9 ± 1.1, 5.6 ± 0.06, and 16.4 ± 0.28%, respectively. Additionally, the pro-oxidant activity of shionone in ferric bleomycin in induced DNA damage was found to be 1.60 ± 0.34% of vitamin C. Among all antioxidant assays, shionone was not among the top-performing compounds, and it has not had effective antioxidant activity. The study suggested that the antioxidant property of AT may be majorly attributed to other compounds, such as quercetin and kaempferol present in AT [39].

### 4.7. Expectorant and Antitussive Activities of Shionone

AT has been used as the treatment of cough and dispelling phlegm in China since ancient times. Hence, the active fraction of AT and the main triterpenoid component of AT (i.e., shionone) were studied for both expectorant and antitussive activities [40]. ICR male mice were used as the animal model in the study in both experiments. In expectorant activities, phenol red secretion was studied in different study groups, which included the control, shionone treatment, positive drug treatment, and AT extract treatment groups. The expectorant activity of shionone in terms of the secretion of phenol red was lower than the AT extract and positive drug groups. The antitussive activity of shionone in terms of the latent period and the frequency of cough was not significant. The study suggested that shionone may not have a significant role in the expectorant and antitussive activity of AT root extract, and the other components of the extract may be further studied for these activities [40].

### 4.8. Anti-Colitis Activity of Shionone

Colitis is an important inflammatory bowel disease (IBD), which is a major public health concern owing to the growing number of widespread cases [41]. Colitis is a chronic disease of the digestive system that is characterized by the inflammation of the inner lining of the colon [42].

The anti-colitis activity of shionone was discovered in animal models through dextran sodium sulfate (DSS)-induced colitis in BALB/c mice. After the treatment, both shionone at 25 and 50 mg/kg-administered groups showed a reduction in the inflammation of colon tissue with H&E staining experiments. The major factor of inflammasome NLRP3 was also found to be reduced in both immunohistochemistry and Western blot analysis of colon tissue in both shionone-administered groups [43]. Other important markers, such as caspase-1 and IL-1β, were also found to be inhibited with shionone treatment (Table 2 and Figure 2). The study revealed that the administration of shionone may reduce local inflammation in the colon, which supports the role of the anti-inflammatory activity of shionone for its anti-colitis effects. Future studies may be required to unveil the molecular action mechanisms of shionone for anti-colitis activity and its development as a therapeutic agent [43].

### 4.9. Anti-Parkinson’s Disease Activity of Shionone

Parkinson’s disease (PD) is the second most common neurodegenerative disease after Alzheimer’s disease (AD). Its global incidence increases with time, which again demands effective treatment [44]. Neuroinflammation is considered an important pathological factor associated with PD, which supports the study of anti-inflammatory compounds for its treatment and prevention [45].

**Table 2:** In vivo pharmacological activities of shionone.

Activity/Probable Mechanism	Model and Dose	Method	Major Findings	Reference
Lung protective activities	Female BALB/c mice and 50 mg/kg orally twice a day	Intranasal infection by *S. pneumoniae* and histopathology analysis	Structurally intact alveolar tissue↑, inflammatory cell infiltration↓, lung colonies↓, and lung injury↓	[19]
	Male ICR mice and 10, 50, and 100 mg/kg intragastrically	CLPS to create the sepsis model	The survival percent↑	[20]
		Histopathology of lung H&E staining	Thickening of alveolar walls↑, infiltration of inflammatory cells↓, pulmonary edema↓, and pulmonary wet/dry ratio↓	
		ELISA and the myeloperoxidase activity assay	Serum level of TNF-α↓, IL-6↓, and IL-1β↓, BALF protein↓, and lung MPO activity↓	
		Flow cytometry	Percentage of cell population neutrophils and macrophages in BALF↓	
		WB and immunohistochemistry staining	CD16/32↓, iNOS↓, CD206↑ and Arg1↑ In ECM1/STAT5/NF-κB, phosphorylation of STAT5↑, ECM1 expression↓, and the phosphorylation of NF-κB↓ and ECM1 expression↓	
Anti-kidney injury	C57BL/6 mice and 50 and 100 mg/kg	CLPS for acute kidney injury and H&E staining	Survival percentage↑, inflammatory cell infiltration↓, vacuolation↓, blood urea nitrogen↓, and serum creatinine↓	[26]
		ELISA	IL-6↓, IL-1b↓, IL-12↓, and TNF-α↓	
		WB	CD16/32↓, iNOS↓, p-NF-kB/NF-kB↓, ECM1↓ CD206↑, Arg1↑, and p-STAT5/STAT5↑	
Anti-IC activity	Female SD rats and 50 mg/kg 100 mg/kg gavage	IC-induced inflammation and NLRP3 inflammasome activation through cyclophosphamide	Bladder wet weight↓, edema score↓, hemorrhage score↓, and histopathological score↓	[16]
		Protein expression in the bladder by WB	Expression of NF-κB↓, GSGMD-N↓, NLRP3↓, ASC↓, pro-caspase-1↓, and cleaved IL-1β↓	
		Immunofluorescence analysis	Expression of NLRP3↓ NLRP3	
Anti-UTI	Female SD rats and 100 mg/kg and 200 mg/kg through gavage	UPEC solution pushed into the bladder for the animal UTI model and the positive control drug levofloxacin	Congestion↓ and edema↓ in the bladder, infiltration of inflammatory cells↓ in large submucosal and mucosa, and thickening of the bladder↓	
		Electron microscopy of superficial cystic tissue	Lysosomes↑ and vesicles↑	
		Bacterial colony counts in bladder homogenate	*E. coli* in bladder tissue ^#^	
		ELISA assay	IL-1β↓, IL-6↓, and TNF-α↓	
Expectorant and antitussive activity	ICR male mice and 80 mg/kg once daily for 3 consecutive days	Secretion of phenol red in the trachea and parts of the bronchi. NH_4_Cl (250 mg/kg) as a positive control	Secretion of phenol red↑ (by 11.7% ^#^)	[40]
		Cough frequency and the latent period were studied. The positive control was pentoxyverine (17.5 mg/kg)	No significant effects on the latent period and the frequency of cough	
		Anti-inflammatory activity by the mouse ear welling model	Ear edema↓ (by 11.3% ^#^)	
Anti-colitis activity	BALB/c mice and 25 and 50 mg/kg for two weeks	DSS-induced colitis and H&E stain of the colon tissue	Colonic inflammation ↓	[43]
		Immunohistochemistry and the WB assay of the colon tissue	NLRP3↓, caspase-1↓, and IL-1β↓	
Anti-PD activities	Male 57BL/6J mice and 50 mg/kg/day for 7 consecutive days	Intraperitoneal injection of neurotoxin MPTP	Mouse rod rotation and pole climbing experiments	[46]
		Morphological analysis of TH expression at the substantia nigra site by immunohistochemistry	TH positive neurons↑	
		Striatum part dopamine content through the HPLC-based method	Increase the dopamine level in the striatum↑	
Anti-DN	C57BL/6 wild type mice and 25 mg/kg for eight weeks	DN was induced through single nephrectomy and streptozotocin administration	Body weight ↑, kidney index↓, urine volume↓, urine protein↓, serum creatinine↓, serum urea nitrogen↓	[28]
		H&E staining	Local fibrosis lesions of glomeruli↓	
		Immunohistochemistry of kidney tissues (glomeruli)	NLRP3↓ and IL-1β↓	

CFUs, colony forming units; CLPS, cecal ligation and puncture surgery; DN, diabetic nephropathy; DSS, dextran sodium sulfate; ELISA, enzyme-linked immunosorbent assay; H&E, hematoxylin and eosin staining; HPLC, high-performance liquid chromatography; IC, interstitial cystitis; MPTP, N-methyl-4-phenyl-l,2,3, 6-tetrahydropyridine; PD, Parkinson’s disease; RTPCR, reverse-transcriptase polymerase chain reaction; TH, tyrosine hydroxylase; UTI, urinary tract infection; WB, Western blot; ^#^ not significant; ↑: up-regulation; ↓: down-regulation.

The anti-PD activity of shionone was studied in the mouse model of PD. Male 57BL/6J mice were selected for the study and intraperitoneal injection of N-methyl-4-phenyl-l,2,3, and 6-tetrahydropyridine (MPTP) was used to create the PD model. After 7 days of administration, shionone significantly improved the motor dysfunction of the animals in both mouse rod rotation and pole climbing experiments [46]. Further, in an immunohistochemical study substantia nigra part of the mouse brain showed an increase in tyrosine hydroxylase (TH)-positive neurons in shionone-treated groups (Table 2 and Figure 2). Additionally, the dopamine level of the striatum part of the brain also increased in the shionone treatment group. The study suggested that the significant improvement in motor dysfunction in the PD mouse model may be due to the increase in TH-positive neurons and dopamine content [46]. These results warrant further studies to be conducted on different models to validate the findings before clinical studies.

### 4.10. Pharmacokinetic Studies of Shionone

Shionone is one of the major bioactive triterpenoids of AT and is believed to be responsible for major pharmacology properties of AT. Therefore, the pharmacokinetics of shionone were analyzed in different studies along with other components of AT. Wistar rats were used as an animal model for the study, and researchers have used liquid chromatography–tandem mass spectrometry (LCTMS) to study the pharmacokinetics of shionone and epi-friedelinol in the plasma of the animals after the oral administration of AT [47]. The lower limit of quantitation of shionone was found to be 7.60 ng/mL after 12 h in the experiments, which indicated sufficient sensitivity for the investigation of the pharmacokinetic behavior of shionone. The maximum plasma concentration (Cmax), the time at which the Cmax is achieved (Tmax), the area under the plasma concentration time curve (AUC0–t), the area under the plasma concentration time curve to time infinity (AUC0–∞), and the elimination of the half-life (t1/2) of shionone were found to be 409.90 ± 61.02 ng/mL, 1.23 ± 0.52 h, 1474.36 ± 211.31 (ng/mL) h, 1504.73 ± 205.12 (ng/mL) h, and 1.75 ± 0.19 h, respectively. Recently, the ultra-high-performance liquid chromatography–tandem mass spectrometry (UHPLCTMS) method was utilized to study the pharmacokinetics of shionone along with fourteen other important compounds present in the AT extract. In the study, the pharmacokinetics of the selected compounds were studied for raw and honey-processed AT after oral administration in male SD rats. The important parameters for raw AT such as Cmax, Tmax, AUC0–t, AUC0–∞, and t1/2 of shionone were found to be 3134.61 ± 548.07 ng/mL, 6.33 ± 0.82 h, 45,286.04 ± 2265.18 (ng/mL) h, 45,372.62 ± 2260.78 (ng/mL) h, and 5.31 ± 0.74 h, respectively. Similarly, for honey-processed AT, the Cmax, Tmax, AUC0–t, AUC0–∞, and t1/2 of shionone were found to be 1929.37 ± 483.99 ng/mL, 5.67 ± 1.51 h, 32,320.11 ± 8225.70 (ng/mL) h, 32,524.44 ± 8285.98 (ng/mL) h, and 7.28 ± 1.67 h. The study revealed that the AUCs of shionone in the raw AT were significantly higher than the honey-processed AT [48]. These pharmacokinetics parameters would be helpful for the formulation of dosages of shionone in clinical experiments and its further development as a drug candidate [48].

## 5. Discussion

Encouraging results in recent studies of the different biological activities of shionone augmented the efforts of the progress of shionone as a therapeutic and/or supplement against different diseases, which also resulted in several recent patent applications [22,28,43,46]. Numerous studies suggested that the anti-inflammatory activities of shionone may be the major reason for the protective effects on multiple organs, which can explain its multiple pharmacological properties. The protective effects on lungs, including anti-COPD activities, were studied due to the usage of parent AT extract in ancient Chinese medicine to treat cough and phlegm. In these lung protective studies, shionone suppressed the inflammatory response, marker genes, and pathways, such as pulmonary edema, TNF-α, IL-6, and IL-1β and ECM1/STAT5/NF-κB, which again support the anti-inflammatory properties of shionone [19,20]. Apart from anti-inflammatory activity, shionone also protects the lungs from pneumolysin through the inhibition of its expression and oligomerization [19]. Similarly, in the protection against UTIs, shionone not only inhibits *E. coli* but also reduces the inflammation-associated markers, such as IL-1β, IL-6, and TNF-α, like in previous studies [26]. In anti-IC activity, shionone could alleviate inflammation and pyroptosis in the bladder by suppressing the gene and protein expression of NF-κB, NLRP3, ASC, pro-caspase-1, caspase-1, GSDMD, and GSDMD-N in vivo studies, which was further reinforced in in vitro cell studies (Table 1 and Table 2). Studies support the anti-inflammatory activity of shionone due to its anti-IC effects and its drug candidature for IC. Shionone also protects the kidney by targeting the ECM1/STAT5 pathway and suppresses inflammatory factors such as IL-6, IL-1b, IL-12, and TNF-α in both in vitro and in vivo experiments. Similarly, in the case of DN, the positive effects of shionone on the kidney were observed with a reduction in inflammatory markers and genes (Table 2). Furthermore, the anti-colitis activity of shionone was also observed with a reduction in inflammation and inflammatory factors such as NLRP3, caspase 1, and IL-1β in the colon. It can be concluded that the protective activities of multiple organs of shionone are majorly based on its anti-inflammatory potential, which suggests that further research to study the protective effect of shionone on other organs, such as the liver and other inflammation-associated diseases, like inflammatory bowel diseases, such as Crohn’s disease, is needed [49]. Importantly, shionone administration also improved in the PD model of mice with an increase in the content of dopamine level and tyrosine hydroxylase (TH)-positive neurons in the striatum and substantia nigra part of the brain, respectively. These results in the brain give hope for treating devastating neurodegenerative disorders. In previous studies, a reduction in inflammatory markers with shionone administration in different organs strongly suggests studying the anti-inflammatory status in the brain with shionone treatment, as it may also be responsible for improvement in neurodegenerative PD [50]. These future studies may pave the path for the development of shionone against not only PD but also other neurodegenerative diseases, such as AD.

The potent antioxidant activity of shionone was not observed in different experiments, which discourages future studies of the antioxidant potential of shionone, but the enzymatic antioxidant activity of shionone, which is not studied, may be pursued, as per the requirement of the studies. Similarly, strong expectorant activity is also not observed in the case of shionone, which indicates that the role of other components of AT may be more important for this activity. Although the anti-cancer potential of shionone was only studied against breast cancer, the anti-cancer activity of shionone may be the prospective activity of shionone, which must be developed further as it not only specifically kills the cancer cells in the study but also suppresses the invasion and migration of cancer cells. The important signaling pathways found to be targeted in the studies were MEK/ERK and STAT3. The anti-inflammatory activities of shionone in other studies, specifically the inhibition of important target genes, such as NF-κB, can also contribute to its anti-cancer activity, as it is also the established target for anti-inflammation-based anti-cancer activities. Currently, the anti-cancer status of shionone is in its initial phase; it is suggested to study the anti-cancer potential of shionone against breast cancer in in vivo experiments before clinical studies. Considering the effect of shionone on important targets in multiple cancers, such as NF-κB and STAT3 [5,7], studies are also suggested for other cancers in the near future. It may also be suggested that the genes/markers associated with diseases found to be targeted in more than one study may be considered highly important targets of shionone (Figure 2). These important target genes are crucially associated with inflammation, which strongly supports the development of shionone against different diseases where inflammation is the important aspect of the etiology, i.e., AD, cancer, obesity, etc. [51,52].

Initial pharmacokinetics studies also support shionone as the therapeutic candidate of AT. These studies provided the basis to design doses in further in vivo and clinical experiments and also suggested searching for alternate administration strategies in future studies. Still, more specific pharmacokinetic studies on shionone may be required to develop its dosage form in different diseases according to requirements. Now, it can be stated that shionone is a potential compound against various diseases and in most diseases, the anti-inflammatory effect of shionone may be the major reason for therapeutic activities. However, according to the activity, its development as a therapeutic is in different stages that may be pursued in the proposed steps to fill the suggested gaps in the present review accordingly.

## Data Availability

Data are contained within the article.
